# The genetic association between hyperthyroidism and heart failure: a Mendelian randomization study

**DOI:** 10.3389/fendo.2024.1344282

**Published:** 2024-04-12

**Authors:** Jun Liu, Gujie Wu, Shuqi Li, Lin Cheng, Xinping Ye

**Affiliations:** ^1^ Department of Health Management, The First Hospital of Hunan University of Chinese Medicine, Changsha, Hunan, China; ^2^ Department of Endocrinology and Metabolism, Zhongshan Hospital, Fudan University, Shanghai, China; ^3^ Department of Thoracic Surgery, Zhongshan Hospital, Fudan University, Shanghai, China; ^4^ Regenerative Medicine Institute, School of Medicine, National University of Ireland (NUI), Galway, Ireland

**Keywords:** thyroid disease, hyperthyroidism, heart failure, all-cause heart failure, two-sample Mendelian randomization (MR), SNPs

## Abstract

**Background and aims:**

Hyperthyroidism is an endocrine disease with multiple etiologies and manifestations. Heart failure (HF) is a common, costly, and deadly medical condition in clinical practice. Numerous studies have suggested that abnormal thyroid function can induce or aggravate the development of heart disease. However, no study has demonstrated a causal relationship between hyperthyroidism and heart failure. Therefore, the purpose of this study was to explore the causal link between hyperthyroidism and HF.

**Methods:**

Summary data for genetically predicted hyperthyroidism were obtained from a genetic association study. The data examined for genetically determined all-cause heart failure came from 218,208 individuals from the FinnGen Consortium. Two-sample Mendelian randomization (MR) analysis was used to estimate the causal link between hyperthyroidism and heart failure. Statistical analyses were conducted using the inverse variance-weighted, weighted median, simple median, weighted mode, MR-PRESSO (number of distribution = 5000), MR-Egger, and leave-one-out.

**Results:**

The results of the inverse-variance weighted analysis indicated a causal association between hyperthyroidism and an increased risk of all-cause heart failure (IVW: β=0.048, OR=1.049, 95%CI: [1.013 to 1.087], P=0.007). Similarly, the weighted median approach demonstrated a positive correlation between hyperthyroidism and all-cause heart failure (OR=1.049, [95% CI, 1.001-1.100]; P=0.044). Additionally, no horizontal pleiotropy or heterogeneity was observed. The leave-one-out analysis revealed that the majority of the SNP-driven associations were not influenced by a single genetic marker.

**Conclusion:**

Our study observed a causal relationship between hyperthyroidism and all-cause heart failure. Hyperthyroidism may associate with heart failure genetically.

## Introduction

Thyroid hormones are endocrine hormones that are synthesized and secreted by the thyroid cell. Circulating thyroid hormones can act on a wide range of cells and are necessary for growth and energy metabolism. Hyperthyroidism occurs when an excess of thyroid hormone is synthesized and secreted. Previous surveys have shown the prevalence of hyperthyroidism to be 0.8% in Europe and 1.3% in the USA (1, 2). An observational study based on the Chinese population reported that the incidence of hyperthyroidism in iodine-sufficient areas is about 1.2% (3), meaning that one in a hundred people is hyperthyroid.

According to the presence or absence of clinical symptoms, hyperthyroidism is classified into overt or subclinical types. Symptoms of overt hyperthyroidism primarily result from excessive hormones, leading to hypermetabolism and symptoms of sympathetic nerve excitation, such as palpitations, sweating, anxiety, and weight loss (4). Atrial fibrillation is a complication of hyperthyroidism and is considered as an independent risk factor for congestive heart failure (5), while heart failure is the primary cause of cardiovascular events (6). Additionally, thyrotoxic periodic paralysis, a harmful complication more prevalent among East Asian individuals than North Americans (0.2% versus 2%) (7), is characterized by muscle paralysis, acute hypokalemia, and thyrotoxicosis (8), and can lead to severe arrhythmia or muscle weakness.

Heart failure (HF) is a cardiac disorder caused by heart dysfunction and is one of the common diseases in clinical practice. Research findings indicate that the prevalence of HF is approximately 1–2% in the developed countries, while the figures are higher in developing areas (9). According to a report from the American Heart Association (AHA), the percentage of the population with HF is 1.5%, 6.6%, and 10.6% for men in different age groups (40-59, 60-79, and ≥80 years), respectively. The corresponding percentages for women are approximately 1.2%, 4.8%, and 13.5% (10).

HF can significantly increase the hospitalization and mortality rates of patients. A 4.7-year follow-up study showed that patients with HF often require hospitalization, with up to 4,359 hospitalizations occurring among 1,077 patients, averaging 4 hospitalizations per person (11). More than 70% of these hospitalizations occurred among adults aged ≥65 years (12). Stewart’s study, which enrolled 16,224 men and 14,842 women admitted to the hospital for heart failure, myocardial infarction, or cancer, found that male patients lose about 6.7 years of life expectancy per 1,000 people, and 5.1 years per 1,000 for women. The mortality rate for heart failure was found to be higher than that of many cancers (13). An observational study suggested that the mortality rate of HF patients during five years of hospitalization is over 65% (14). Additionally, the prognosis is worse for individuals hospitalized with HF. Studies have indicated that the mortality rate of patients within one month of hospitalization was 10.8%, which was three times higher for patients within one year of hospital admission (15, 16). With the growth of the population, the aging problem, and the prevalence of other cardiac diseases, heart failure will increasingly present a severe challenge.

A study found that 6% of patients with hyperthyroidism were diagnosed with heart failure as the initial symptom (5). Numerous studies have indicated a strong association between hyperthyroidism and the development of heart failure. It was reported that thyroid hormones in the bloodstream can impact the systolic function of the left ventricle, thereby influencing cardiac ejection fraction (EF) and output (17). In overt hyperthyroidism, there is a significant increase in the incidence of left ventricular (LV) hypertrophy, as well as an increase in left ventricular ejection fractions (LVEFs) and contractility (18, 19). Similarly, subclinical hyperthyroidism is closely related to heart disease. Baris found that the risk of heart failure events was higher in patients with subclinical thyroid dysfunction (20). A previous study demonstrated that subclinical hyperthyroidism causes greater impairment of cardiac function than overt hyperthyroidism (21). However, there are no studies that have examined the causal relationship between hyperthyroidism and heart failure. Although two studies have explored the causal relationship between thyroid function and cardiac-related diseases, the thyroid hormone concentrations in the population they enrolled were mainly in the normal range and cannot represent hyperthyroidism (22, 23). Therefore, we utilized GWAS data specifically detecting genes for hyperthyroidism and hypothesized that a causal link exists between hyperthyroidism and heart failure, and aimed to explore this through a Mendelian study.

## Methods

### Study design

**Figure 1:**
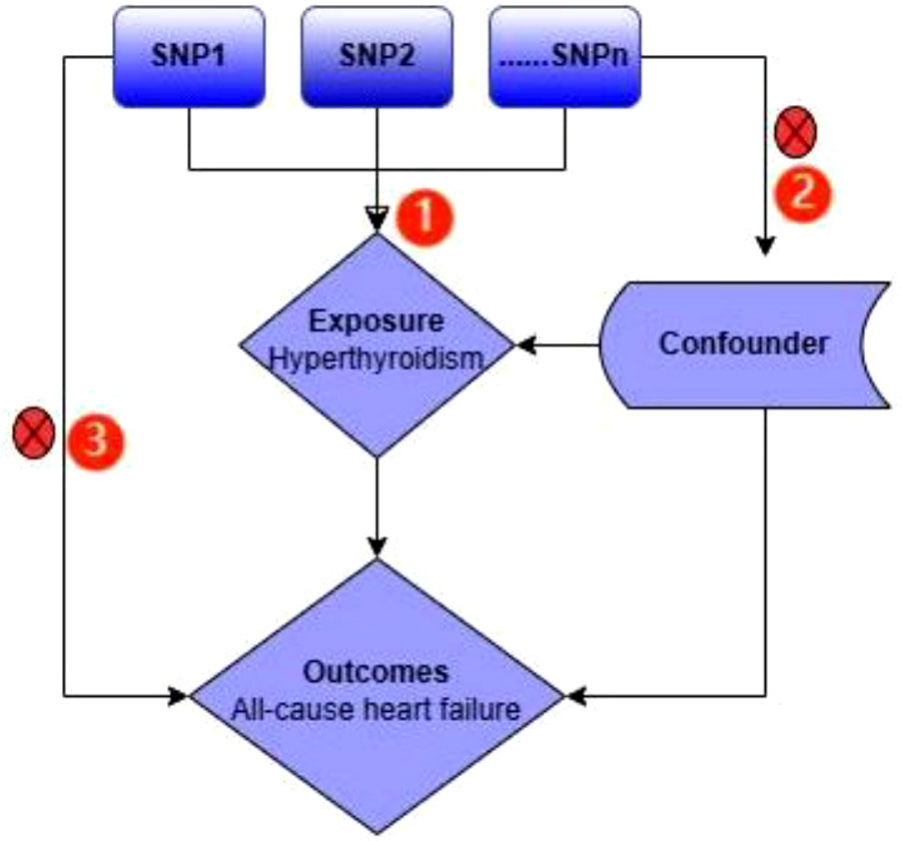
An overview of the study design. The serial number represents 3 assumptions; SNP, single‐nucleotide polymorphism.

In this study, all-cause heart failure were the outcomes. We adopted two-sample Mendelian randomization (MR) study to evaluate the causal effect of hyperthyroidism on them (**Figure 1**). The MR design was guided by three key assumptions: (1) genetic variants chosen as the genetic instrumental variables must be powerfully related to hyperthyroidism; (2) no link should be found between genetic variants and any confounding factors, and (3) genetic variants must be associated with outcomes only through hyperthyroidism but not via any other causal pathway (24). Our data were mainly based on independent genome‐wide association studies (GWAS).

### Data sources

Genetic variants of hyperthyroidism were collected from a European cohort comprising 460499 participants (case=3557, control=456942) (25). A summary dataset of all-cause heart failure was derived from the FinnGen study (26). All of these data were obtained from the IEU Open GWAS project (https://gwas.mrcieu.ac.uk/, updated to 2023.09.06, N= 42354), which has collected a great deal of genetic data from thousands of studies (**Table 1**).

**Table 1:** Details of studies and datasets used in the study.

Exposure /Outcomes	GWAS ID	Sample size	Year	Population
hyperthyroidism	ebi-a-GCST90018860	460499	2021	European
All-cause heart failure	finn-b-I9_	218208	2021	European
	HEARTFAIL_ALLCAUSE			

NA, not applicable.

### Instrumental variable selection

We selected genetic variants of hyperthyroidism that were eligible for screening (P<5×10-7, linkage disequilibrium (LD) r^ (2) <0.001, and clumping distance =10000kb) as the instrumental variables (IVs) for MR analysis when all-cause heart failure was the outcome (27). F statistics of all IVs were greater than 20 here, and “weak IV” was excluded.

### Statistical analysis

We applied Two-sample Mendelian randomization (MR) analysis to evaluate the causal link between hyperthyroidism and heart failure. The inverse variance-weighted (IVW), weighted median, Simple median, Weighted mode, MR-Egger, MR-PRESSO (NbDistribution = 5000), and Leave-one-out methods were employed for formal analysis. Studies have shown that the IVW method was slightly more precise than the others (28). For this reason, we have taken the results of the IVW as the primary evidence and the results of the other methods as complementary evidence.

To investigate whether the causal result driven by a single SNP existed, a leave-one-out analysis was performed. All statistical analyses were performed using the R packages: Two-sample MR (version 0.5.7) (29) and MR-PRESSO (version 1.0) (30).

### Heterogeneity and horizontal pleiotropy

Cochran’s Q statistics and I2 index were adopted to assess the heterogeneity in our analysis, and the p-value of Q statistics <0.05 or I^2^%≥31% represents the presence of heterogeneity. Finally, we used the Egger regression intercept to estimate the horizontal pleiotropy.

## Result

### Primary MR analysis of hyperthyroidism and all-cause heart failure

**Figure 2:**
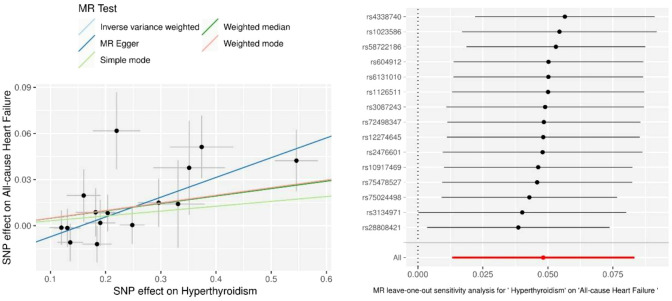
Scatter plots and Leave-one-out analysis of All-cause heart failure. Results are shown for different Mendelian randomization (MR) methods, including inverse variance weighted (IVW), simple median, weighted median, weighted mode, and MR-Egger.

Only 16 genetic variants were clumped and selected as the eligible instrumental variables from the summary data of hyperthyroidism. The SNP named rs385863 was removed from the clumped variants, leaving 15 SNPs for MR analysis (**Table 2**). In the Mendelian randomization results for the causal effects of hyperthyroidism on All-cause heart failure, the P-values of the MR Egger, Weighted median, and Inverse variance weighted were all <0.05 (P=0.008, 0.044, 0.007, respectively) (**Table 2**), supporting a causal relationship between hyperthyroidism and All-cause HF. The results of the horizontal pleiotropy and heterogeneities test did not reveal any pleiotropy or heterogeneities in this analysis (p>0.05) (**Tables 3**, **4**), and the P-value of Global Test in MR-PRESSO (Nb Distribution = 5000) (P= 0.394) further supports it. A scatter plot was included to better validate our findings (**Figure 2**). Finally, a forest plot was created to vividly reveal the results of the sensitivity analysis (**Figure 2**).

**Table 2:** Mendelian randomization results for the causal effects of hyperthyroidism on All-cause heart failure.

	Method	N SNP	β	OR	95% CI	P value
All-cause heart failure	MR Egger	15	0.129	1.137	1.049 to 1.233	0.008
	Weighted median	15	0.048	1.049	1.001 to 1.100	0.044
	Inverse variance weighted	15	0.048	1.049	1.013 to 1.087	0.007
	Simple mode	15	0.032	1.032	0.956 to 1.115	0.435
	Weighted mode	15	0.049	1.051	0.986 to 1.120	0.150

IVW, inverse-variance weighted; MR, Mendelian randomization; OR, odds ratio; CI, confidence interval.

**Table 3:** Results of horizontal pleiotropy in Mendelian randomization studies.

Outcome	Egger regression intercept	Se	P value
All-cause heart failure	-0.020	0.009	0.052

**Table 4:** The heterogeneities were quantified by Cochran Q statistic and I^2^% index.

Outcome	Method	Q	Q_df	P value	I^2^%
All-cause heart failure	MR-Egger	10.89	13	0.620	19.4
	IVW	15.45	14	0.348	9.39
	MR‐PRESSO Global Test	/	/	0.394	/

IVW, inverse-variance weighted. I^2^=(Q−df)/Q.

## Discussion

In the current study, we utilized five methods to test our hypothesis regarding the causal relationship between hyperthyroidism and heart failure (HF) (with all-cause HF as the outcome). The results of the Weighted median and IVW methods both supported our hypothesis (OR=1.049 and 1.049, respectively, all P<0.05), indicating that more severe hyperthyroidism is associated with a higher risk of all-cause HF.

Hyperthyroidism is primarily caused by abnormal thyroid function. Previous studies have demonstrated associations between thyroid function and heart failure. A pooled analysis of 25,390 participants showed an increased risk of heart failure events associated with abnormal thyroid hormone concentrations, especially for TSH levels of ≥10 and <0.10 mIU/L (20). A study performed by Shmuel, which followed up with 5,599 patients with heart failure in the Health Maintenance Organization, found that thyroid stimulating hormone (TSH) levels were predictive of death and associated with poorer clinical outcomes (31). In HF patients with ejection fractions below 35%, the abnormal thyroid function group had a higher death rate compared to the normal group (32). The use of 3,5-diiodothyropropionic acid (DITPA), a TSH receptor agonist, was found to effectively improve cardiac and systemic vascular resistance index (p<0.05) (33). Additionally, two studies have supported the causal link between thyroid hormone levels within the normal range and cardiac-related diseases (22, 23). The results of the IVW method indicate a positive correlation between hyperthyroidism and all-cause heart failure in this analysis.

In general, thyroid hormones can directly or indirectly mediate cardiac function through genomic and nongenomic mechanisms. At the genomic level, T3 combines with a specific nuclear receptor that regulates the expression of several genes with essential physiological roles in the circulatory system (34). Prolonged exposure to elevated concentrations of T3 can upregulate the production of cardiac proteins, eventually leading to the development of myocardial hypertrophy and disorders (35). Thyroid hormones can regulate the expression of genes that modulate the function of the heart, such as the myosin heavy chain (MHC) α gene, the MHC β gene, the Phospholamban (PLN) gene, the Collagen gene, and others (36–38). The non-genomic mechanisms of action of thyroid hormones primarily involve the membrane transport of Na+ and other ions. Thyroid hormone induces rapid alterations in Na+, K+, and Ca2+ channels on cell membranes, altered polymerization of the actin skeleton, and changed intracellular signaling pathways in the cardiac and smooth muscle cells (34). Additionally, they can stimulate the synthesis of NO in the endothelium and subsequently induce vasodilation by activating the phosphatidylinositol 3-kinase (PI3K)/serine/threonine protein kinase (AKT) signaling pathway (39, 40). Thyroid hormones also regulate levels of the sarcoplasmic reticulum calcium-activated ATPase, phospholamban, and myosin heavy chain (α isoforms), all of which contribute to myocardial systolic and diastolic activity (41). In conclusion, both genomic and nongenomic pathways are critically involved in the regulation of cardiac function by thyroid hormones.

Two Mendelian randomization studies have investigated the association between thyroid function and heart failure. Using summary statistics data from multiple studies, Wang found that lower levels of TSH were associated with a lower risk of heart failure (OR=0.82; 95% CI [0.68, 0.99], P< 0.05) (22). Additionally, a study involving 105,224 individuals indicated that TSH concentrations below the population median (1.53 mIU/L) were observationally and genetically associated with an increased risk of AF, MI, and AVS, while the results of MR analysis were non-significant (all p>0.05) (23). Thyroid hormone concentrations in both studies were within normal ranges, whereas our study used hyperthyroidism, a disease characterized by abnormal thyroid hormone levels, as the exposure factor. Our study demonstrated a causal relationship between hyperthyroidism and all-cause heart failure, suggesting that hyperthyroidism may contribute to the development of all-cause heart failure genetically. With the continuous in-depth research on the pathogenic genes of diseases, in the future, after the genetic relationship between hyperthyroidism and heart failure is further clarified, it may be possible to assess the risk of heart failure early by detecting the genes of patients with hyperthyroidism, so as to prevent the occurrence of heart failure and improve the outcome of patients.

There are several limitations in this study. First, we cannot completely rule out the pleiotropy of SNPs acting on outcome variables, although the pleiotropy test was negative in this case. Additionally, MR analysis can only detect a linear relationship between exposure and outcome factors. And our study did not explore specific molecular mechanisms regarding hyperthyroidism and heart failure, and larger RCTs are warranted to verify our conclusions. The study lacks of a clear definition of overt and subclinical hyperthyroidism because the data used in this study could not distinguish between the two groups of patients separately. This study has not been possible to assess the possible correlation between thyrotoxicoses without thyroid hyperfunction and heart failure.

Ultimately, our findings demonstrate a causal relationship between hyperthyroidism and an increased risk of all-cause heart failure at a genetic level, indicating that hyperthyroidism may be associate with heart failure genetically.

## Data availability statement

The original contributions presented in the study are included in the article/supplementary material. Further inquiries can be directed to the corresponding authors.

## Author contributions

JL: Writing – original draft. GW: Data curation, Software, Writing – review & editing. SL: Formal analysis, Methodology, Writing – review & editing. LC: Writing – review & editing. XY: Writing – review & editing.
